# Refining a Protocol for Faecal Microbiota Engraftment in Animal Models After Successful Antibiotic-Induced Gut Decontamination

**DOI:** 10.3389/fmed.2022.770017

**Published:** 2022-02-09

**Authors:** Nadia Amorim, Emily McGovern, Anita Raposo, Saroj Khatiwada, Sj Shen, Sabrina Koentgen, Georgina Hold, Jason Behary, Emad El-Omar, Amany Zekry

**Affiliations:** ^1^Microbiome Research Centre, St. George and Sutherland Clinical School, UNSW Sydney, Sydney, NSW, Australia; ^2^Department of Gastroenterology and Hepatology, St. George Hospital, Sydney, NSW, Australia

**Keywords:** antibiotics, faecal microbiota transplant, microbiome, gut decontamination, gut engraftment

## Abstract

**Background:**

There is mounting evidence for the therapeutic use of faecal microbiota transplant (FMT) in numerous chronic inflammatory diseases. Germ free mice are not always accessible for FMT research and hence alternative approaches using antibiotic depletion prior to FMT in animal studies are often used. Hence, there is a need for standardising gut microbiota depletion and FMT methodologies in animal studies. The aim of this study was to refine gut decontamination protocols prior to FMT engraftment and determine efficiency and stability of FMT engraftment over time.

**Methods:**

Male *C57BL/6J* mice received an antibiotic cocktail consisting of ampicillin, vancomycin, neomycin, and metronidazole in drinking water for 21 days *ad libitum*. After antibiotic treatment, animals received either FMT or saline by weekly oral gavage for 3 weeks (FMT group or Sham group, respectively), and followed up for a further 5 weeks. At multiple timepoints throughout the model, stool samples were collected and subjected to bacterial culture, qPCR of bacterial DNA, and fluorescent *in-situ* hybridisation (FISH) to determine bacterial presence and load. Additionally, 16S rRNA sequencing of stool was used to confirm gut decontamination and subsequent FMT engraftment.

**Results:**

Antibiotic treatment for 7 days was most effective in gut decontamination, as evidenced by absence of bacteria observed in culture, and reduced bacterial concentration, as determined by FISH as well as qPCR. Continued antibiotic administration had no further efficacy on gut decontamination from days 7 to 21. Following gut decontamination, 3 weekly doses of FMT was sufficient for the successful engraftment of donor microbiota in animals. The recolonised animal gut microbiota was similar in composition to the donor sample, and significantly different from the Sham controls as assessed by 16S rRNA sequencing. Importantly, this similarity in composition to the donor sample persisted for 5 weeks following the final FMT dose.

**Conclusions:**

Our results showed that 7 days of broad-spectrum antibiotics in drinking water followed by 3 weekly doses of FMT provides a simple, reliable, and cost-effective methodology for FMT in animal research.

## Introduction

The human gut harbours trillions of microbes which include bacteria, archaea, viruses, and eukaryotes. Bacteria and archaea make up most of the gut microbiome, and studies have demonstrated a role for the gut microbiota in influencing disease processes in many organs (1, 2). Unsurprisingly, alterations in the richness, diversity, composition, and function of the gut microbiota have been associated with risks of a broad range of diseases, ranging from gastrointestinal inflammatory and metabolic conditions to neurological, cardiovascular, and respiratory illnesses (2–6). Hence, targeted manipulation of the gut microbiota for personalised nutrition and precision medicine has emerged as an attractive and potential therapeutic strategy (6–9). Indeed, increasing evidence suggests gut manipulation through faecal microbiota transplantation (FMT) has the potential to be beneficial in many diseases (10–12).

Animal models are increasingly used to study the effect of gut microbiome manipulation by FMT on disease pathogenesis, and gut based therapeutic interventions. Although microbial similarity between mice and humans at the phylum level is remarkable, at the genus level 85% of murine sequences represent species that have not been detected in humans (13). “Humanization” of the mouse microbiota is frequently used to overcome this limitation, and it has been shown that mice humanised with different human donors generate a similar microbiome composition and metabolomic profile to that of the donor, with preservation of individual-specific features (14). Thus, for effective translation of therapeutics targeting the gut microbiota, and to explore the mechanisms underlying host-microbe interactions in animals, reconstitution of animals with human donor samples through FMT is key to translational research in this area.

For animal-based FMT studies, recipient animals are required to be either germ free (GF) or depleted of gut bacteria using antibiotics. Germ free mice are considered the gold standard as they are completely devoid of microbes and are therefore perfect candidates for receiving FMT. However, GF animals display an immature and underdeveloped lymphoid system compared with animals living in a conventional microbiological environment (15). In addition, maintaining GF animals requires specialised facilities and may not be feasible in many institutions. Furthermore, GF mice administered FMT also harbour chronic alterations that result in reduced intestinal absorptive function (16). As such, GF animals may not be suitable for all studies, especially when assessing the impact of FMT on host physiology and immunology.

The antibiotic-based approach is usually preferred, as it avoids several issues seen in GF models, allowing mice to undergo normal colonisation and full postnatal development of their immune system (17, 18). However, a consistent protocol is needed for the composition, dose, frequency, length, and mode of antibiotic administration, as highlighted by Kennedy, King (17). Most studies based on antibiotic-induced microbiota depletion of gut use a cocktail comprising of vancomycin, neomycin, ampicillin, and metronidazole. Nonetheless, the duration of antibiotic administration in drinking water varies substantially from 3 days up to 10 weeks (8, 17, 19–21). Prolonged antibiotic use can have an adverse impact on animal health and potentially alter host physiology (19). Hence, it is important to determine the minimum duration of antibiotic treatment required to achieve gut clearance with minimal side-effects.

Similarly, in animal studies, the frequency and duration of FMT administration have varied from a single administration (22), to once daily for 3 days (16), to three doses a week for 3 weeks (23), or once or twice weekly for 4 weeks (22) and up to twice daily for 12 weeks (20). Furthermore, not all studies have analysed microbial composition after completion of FMT administrations to ensure that engraftment of the human microbiota was stable. Consequently, a simple, quick, cost-effective, reproducible, and translational model for reconstituting the animal gut with human gut microbiota is required.

In this study we aimed to address these gaps in the literature, by developing a gut decontamination protocol and determining the minimum duration of antibiotic treatment required to efficiently deplete mice of their commensal gut microbiota. This then enables us to determine the effectiveness of FMT and engraftment stability of human donor microbiota until the end of our follow up period.

## Materials and Methods

### Animals

**Figure 1:**
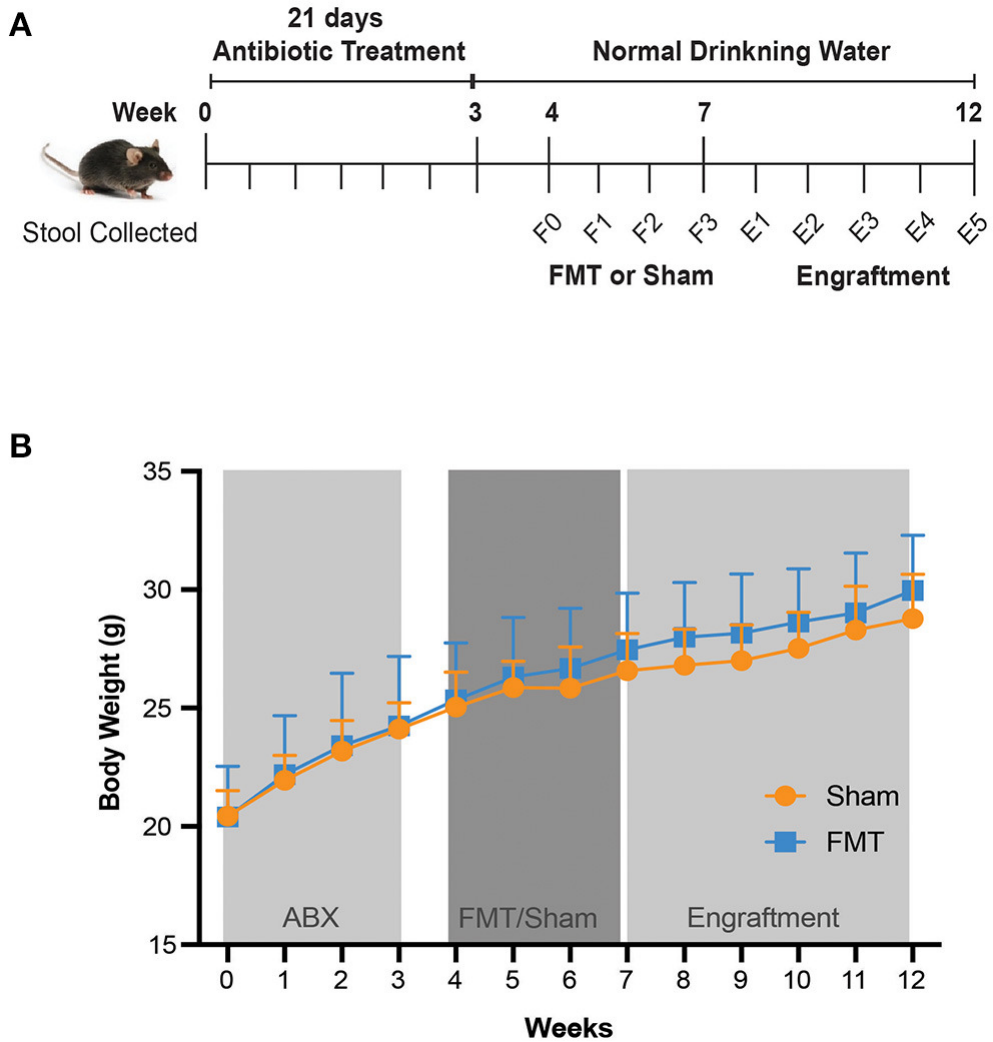
Study design and mouse body weight. **(A)** Study design. From five weeks of age, mice were given antibiotics in drinking water for 21 days. FMT of human donor stool sample (FMT group) or Saline (Sham group) was then orally administered weekly for 3 weeks, followed by a 5 week follow up period. Stool was collected every 3–4 days from day 0 until day 21, and then collected weekly until the completion of the experiment. F0–F3, FMT (Faecal Microbiota Transplant) weeks 0–3; E1–E5, Engraftment weeks 1–5. **(B)** Mouse body weight over the course of 12 weeks, with different treatments. Values shown are average ± SD (*n* =10 mice/group).

Four-week-old male *C57BL/6J* mice were obtained from Australian Biological Resources (NSW, Australia) and allowed to acclimatise for 7 days. Mice were co-housed in a temperature-controlled facility (22–24°C), 12:12 hrs light/dark cycle, with standard irradiated chow (Mouse Maintenance Diet; Speciality Feeds, WA, Australia) and water available *ad libitum*. All mice (*n* = 20) received antibiotic treatment for 21 days. Day 0 corresponded to pre-antibiotics phase. After antibiotic treatment mice were randomised to faecal microbiota transplant (FMT) or Sham groups (*n* = 10 mice per group) (Figure 1A). Animals received either FMT or saline by weekly oral gavage for 3 weeks (FMT group or Sham group, respectively). Mice were monitored and samples collected for a further 5 weeks after oral gavage.

Animal experiments were approved by the Animal Care and Ethics Committee (UNSW Sydney, Ethics ID: 18/79B) and conducted in compliance with the NSW Animal Research Act 1985 and the National Health and Medical Research Council (24).

### Antibiotic Treatment Protocol

Mice received an antibiotic cocktail consisting of Ampicillin 1 g/L, Vancomycin 0.5 g/L, Neomycin 1 g/L, and Metronidazole 1 g/L, in drinking water supplemented with 10% sucrose for 21 days *ad libitum*. This combination of antibiotics is the most commonly used cocktail in literature (17) (reviewed by) and is effective in depleting the microbiome with minimal effects on the morbidity and mortality of the animal (21, 25). The antibiotic solution was changed three times a week. Water intake was monitored throughout the 21 days of antibiotic administration. Following completion of the antibiotic protocol, regular drinking water was provided *ad libitum* for the remainder of the experiment.

### Faecal Microbiota Transplant (FMT) Protocol

Faecal microbiota solution was prepared from fresh human stool sample. Stool was collected and homogenised in phosphate buffered saline (PBS) (0.1 g/mL) within an hour of collection. The mixture was passed through a 100 μm cell strainer, followed by a 40 μm cell strainer, and was stored in 15% glycerol/PBS at −80^o^C. After 21 days of antibiotics, ~0.2 mL (a maximum volume of 10 mL/Kg) of faecal solution (FMT group) or an equivalent volume of Saline (Sham group) was administered to mice via oral gavage once a week for 3 weeks.

### Murine Stool Collection

Stool samples from individual mice (at least three pellets per mouse) were collected aseptically for bacterial culture, fluorescence *in-situ* hybridisation (FISH), qPCR and 16S rRNA sequencing. Stool samples were collected before starting antibiotics on day 0 and every 3–4 days during antibiotic treatment (days 3, 7, 10, 14, 17 and 21). Stool samples were also collected before inoculation of faecal microbiota (F0) and after each of the three doses of FMT which were administrated once a week for 3 weeks (F1, F2, F3, respectively). Post FMT completion, to determine efficiency and stability of gut engraftment over time, stool samples continued to be analysed weekly, for a further 5 weeks, corresponding to the engraftment time points (E)1-E5 (Figure 1A). Stool pellets were weighed individually, and frozen at −80°C until used.

### Stool Culture

To assess bacterial levels in stool pellets, freshly collected pellets were homogenised with 20x volume of PBS, using Qiagen Tissuelyzer II (30 Hz for 1 min). The homogenate was then centrifuged at 300 x *g* for 2 min and the supernatant resuspended in 15% glycerol. A 1:1000 dilution of the stool homogenate was prepared and streaked onto blood agar plates [Columbia Agar Base (Sigma Aldrich Australia) + horse blood (10% vol/vol)] and incubated at 37°C under aerobic, anaerobic and microaerophilic conditions for 48 h before being observed for growth.

### Fluorescent *in-situ* Hybridisation—Fish

A universal 16S rRNA probe for bacteria was used for hybridisation (EUB338). Stool pellets were initially homogenised with 20x volume of PBS. FISH was performed as described previously (26, 27). In brief, samples were fixed in 4% PFA (final dilution of 1:100). For slide preparation, 10 μL of sample was added to a gelatine coated slide and left to air dry at room temperature. Slides were then fixed in 95% ethanol for 10 min. Another 10 μL of fixed sample was added to the slide and dried in a hybridisation oven at 50°C. Bacteria was fixed by soaking the slide for 3 min each in 60, 80 and 95% ethanol. Finally, hybridisation master mix was prepared with 1 μL of probe and 9 μL of hybridisation buffer (0.9 M NaCl; 20 mM Tris pH 8.0; 0.1% SDS). Ten microliters of probe/hybridisation mix were added on top of the cells and hybridised for 90 min at 50°C. Slides were washed in dH_2_O, air dried, and mounted with soft mounting media.

### DNA Extraction

DNA from stool samples was extracted using the PSP Spin Stool DNA plus kit (Stratec Molecular, Berlin, Germany) according to the manufacturer's instructions. Total genomic DNA concentration was determined fluorometrically using Qubit dsDNA broad range assay kit (Qubit 3.0 Fluorometer; Thermo Fisher Scientific, United States).

### Quantitative PCR

Assessment of bacterial load remaining following antibiotic treatment was performed by qPCR. This was performed using PowerUp SYBR Green Master Mix (Life Technologies, TX, USA) with universal 16S primers 926F and 1062R (5'-AAACTCAAAKGAATTGACGG-3' and 5'-CTCACRRCACGAGCTGAC-3', respectively) on the QuantStudio 7 Flex (Life Technologies, CA, USA) with the following cycling conditions: initial denaturation at 95°C for 10 min followed by 40 cycles of 95°C for 15 s and 60°C for 1 min, with a ramp rate of 1°C/s. This was followed by a melt curve analysis from 65 to 95°C at a rate of 0.5°C/s. Each sample was analysed in duplicate. A positive and negative control were run in each plate. Based on the amplification curve, Ct values for each sample were calculated by the instrument when Ct values were below 40.

### 16S rRNA Sequencing

Library preparation and sequencing was performed using 341F and 805R primers (5'-CCTACGGGNGGCWGCAG-3' and 5'-GACTACHVGGGTATCTAATCC-3', respectively) for the 16S V3–V4 rRNA region on the Illumina MiSeq platform with paired 300 bp reads at Ramaciotti Centre for Genomics (UNSW Sydney, Australia).

### Microbiome Analysis

16S rRNA gene forward and reverse reads were imported into Qiime2 (28). The DADA2 pipeline (29) was used for detecting and correcting Illumina amplicon sequences, removal of primers and chimeric reads, and assembly into sequence variants (SV)/operational taxonomic units (OTUs) (30). Taxonomy was assigned using a naïve Bayes classifier trained on the RefSeq database (31). Alpha diversity metrics investigated included Faith's Phylogenetic Diversity (PD), Evenness, Operational Taxonomy Units (OTUs) and Shannon's diversity index which were calculated using qiime2-q2-diversity. Beta-diversity metrics were calculated using Qiime2. Distance metrics calculated included weighted and unweighted UniFrac50 and Bray–Curtis dissimilarity index. Data was visualised using principal coordinate analysis (PCoA) plots and alpha diversity plots generated within R version 4.0.2 using ggplot2 (32) and phyloseq (33). Other packages used included dplyr (34) and qiime2R (35).

### Statistical Analysis

Results are presented as mean ± SD. Statistical analyses for DNA quantification and qPCR were assessed by one-way ANOVA and *post-hoc* Tukey's multiple comparisons test. *P* values of < 0.05 were considered statistically significant. Statistical analyses were performed with Prism v8.4 (GraphPad Software, Inc.).

A Kruskal–Wallis test with Dunn's *post-hoc* test was used to identity individual variations in alpha diversity between treatment groups. Distance based permutation multivariate analysis of variance (PERMANOVA) (36) was performed to test the null hypothesis that there were no differences in the microbial community structure across treatment at a significance level of *P* = 0.05 based on 999 permutations. Resulting *P* values were false discovery rate (FDR) corrected with 0.05 as the cut off. Corrected *P* values are presented as *q*-values.

## Results

### Broad-Spectrum Antibiotic Regimen Did Not Impact General Health of Animals

Throughout the 3 weeks of antibiotic treatment, there were no adverse effects nor animal deaths. Animal well-being was also confirmed by measuring body weight and water intake. All mice gained weight (Figure 1B) and had a stable water intake (Figure S1A), indicating that general health of animals was maintained. Stool weight was unchanged for the first 7 days of antibiotic treatment, with subsequent increase in stool weight observed from day 7 to day 17 compared to day 0 (baseline) (*p* < 0.001) (Figure S1B). At day 21, stool weight had returned to baseline (Figure S1A). No observable change in stool consistency was recorded throughout this period. Importantly, there were no differences in body weight (Figure 1B), water intake (Figure S1A) or stool weight (Figure S1B) when comparing animals that would subsequently receive FMT or Sham, thus providing a stable baseline for subsequent treatment comparisons.

### Broad-Spectrum Antibiotic Regimen Efficiently Decontaminated the Gut After Seven Days

**Figure 2:**
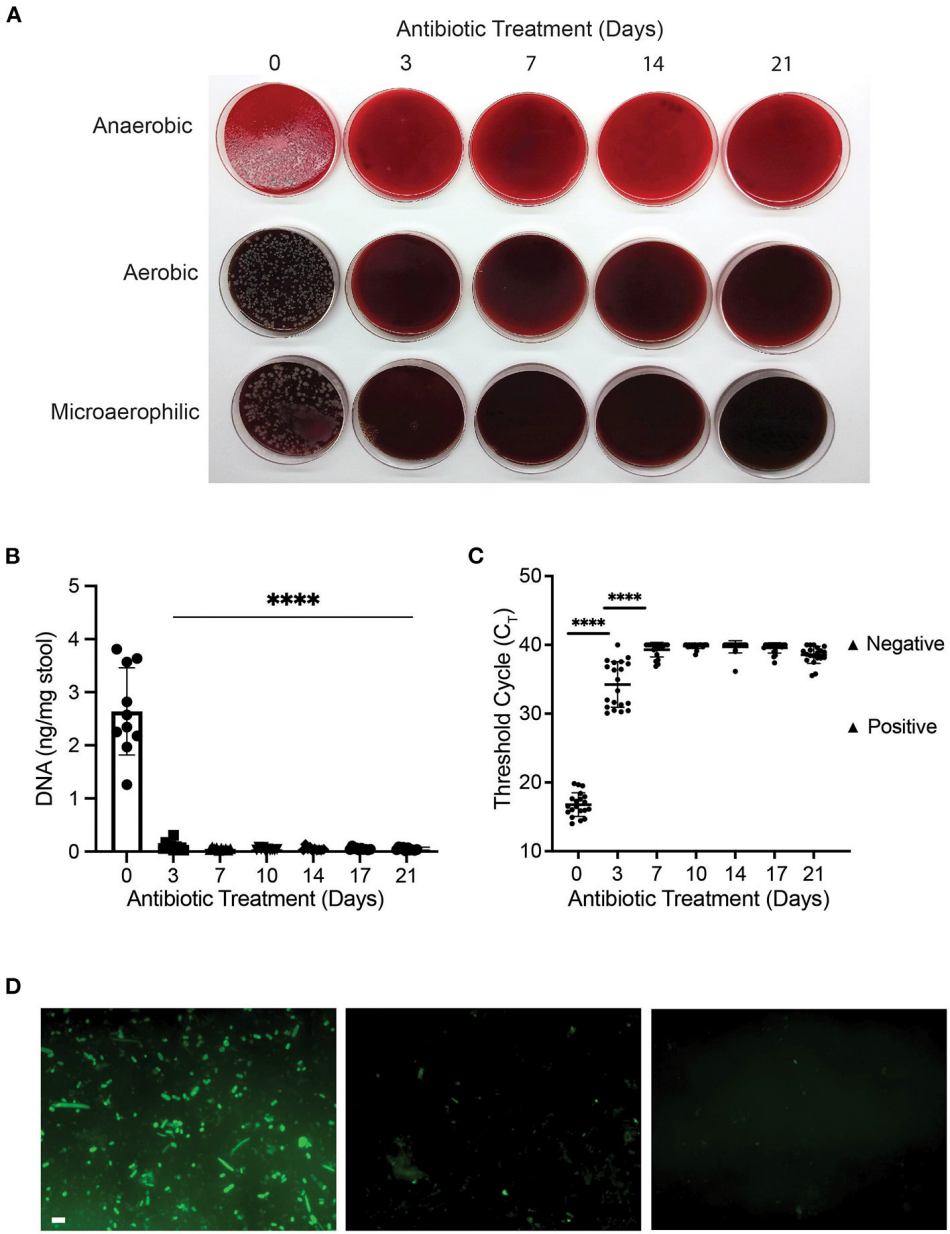
Antibiotic treatment reduced bacterial content of mouse gut after three days. **(A)** Bacterial culture of stool samples on 10% horse blood agar plates; **(B)** DNA concentration (ng/mg of stool) of stool samples during the 21-day course of antibiotic treatment with ampicillin, vancomycin, neomycin and metronidazole (****p < 0.0001, One-way ANOVA); **(C)** Variation in Ct value of 16S rRNA gene amplification during antibiotic treatment (****p < 0.0001, One-way ANOVA). Positive and negative controls for 16S are represented in the graph; **(D)** Representative pictures of fluorescent *in-situ* hybridisation at day 0 (D0; left panel), day 3 (D3; middle panel) and day 7 (D7; right panel) of antibiotic treatment. Scale bar 2 μm.

Next, we determined the optimal antibiotic treatment period for gut decontamination. To evaluate the efficacy of broad-spectrum antibiotic on bacterial depletion, bacterial culture, qPCR and FISH of stool aliquots were undertaken to measure bacterial load. Prior to antibiotic treatment, at day 0 (baseline), measures of high bacterial presence were observed across all assays as expected. Numerous colony forming units (CFUs) were observed on blood agar plates (Figure 2A), DNA concentration was 2.6 ± 0.8 ng/mg stool (Figure 2B), an average qPCR threshold cycle (Ct) of 16.8 was obtained (Figure 2C), and bacteria of various morphologies were confirmed by FISH (Figure 2D).

At day 3 of antibiotic treatment, no CFUs were visible on aerobic and microaerophilic plates, while a dramatic reduction in CFUs was observed on anaerobic plates (Figure 2A). Total stool DNA decreased significantly (96% reduction) when compared to baseline (*p* < 0.00001; Figure 2B), and average Ct values of 34.2 indicates a 100,000-fold reduction in bacterial DNA (*p* < 0.0001; Figure 2C). In addition, limited numbers of fluorescent bacterial cells were observed using FISH assay (Figure 2D), indicating a significant reduction in bacterial presence at day 3 of antibiotic treatment.

From day 7 of antibiotic treatment, there were further reductions in bacterial presence, which remained constant until day 21. At all 5 timepoints between day 7 and day 21 (inclusive), no bacterial growth was observed (Figure 2A), total DNA concentration was reduced by 98% compared to baseline (Figure 2B), average Ct values of 39.3 were equivalent to negative controls (Figure 2C), and no bacterial cells were visible in FISH assay (Figure 2D). Our results showed that broad-spectrum antibiotic cocktail was effective at gut decontamination following 7 days of antibiotic treatment and was maintained thereafter.

### Recolonisation of the Gut After Three Doses of Human Donor FMT Remained Stable for Five Weeks

Following antibiotic gut decontamination, we next investigated the degree of recolonisation by oral administration of either donor faeces (FMT) or saline (Sham) for 3 weeks. There was no impact of FMT or Sham on body weight (Figure 1B).

**Figure 3:**
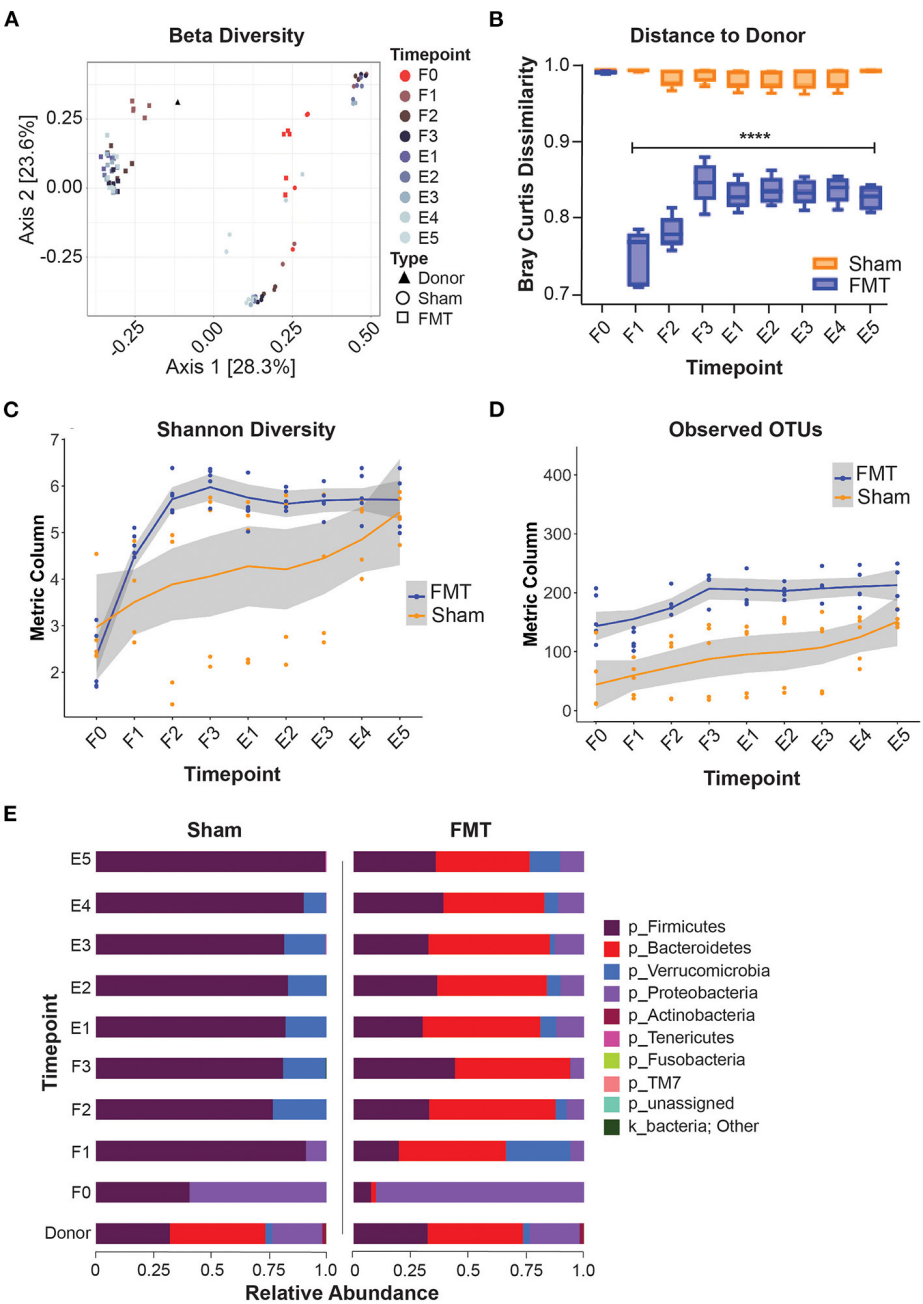
Microbiome composition shift and engraftment after FMT. **(A)** PCoA plot showing beta-diversity of different treatment groups. **(B)** Bray Curtis dissimilarity index comparing distances to donor microbiota. **(C,D)** Alpha-diversity indices during and after FMT. **(E)** taxonomy data (Phylum level) showing successful engraftment of donor microbiota. F0–F3: FMT weeks 0–3; E1–E5, Engraftment weeks 1–5; OTUs, operational taxonomic units.

In animals that received FMT, the microbial composition shifted away from the initial community structure that was present prior to oral gavage (F1–3 vs F0; *q* < 0.05; Figure 3A) and most resembled the donor stool (Bray Curtis dissimilarity index) after the first FMT dose, at F1 (Figures 3A,B). After subsequent FMT doses (F2 and F3), the gut microbiota composition became more dissimilar to the donor stool sample, with subsequent stabilisation during the engraftment period (E1–5) (Figure 3B). Following FMT administration weekly for 3 weeks, the community diversity was increased compared to F0 (*q* < 0.05) and was sustained for a further 5 weeks to the end of the study (Figures 3C,D). Taxonomic profiling of the FMT group at 5 weeks engraftment (E5) confirmed the presence of dominant taxa originating from the donor, including Firmicutes, Bacteroides, Verrucomicrobia and Proteobacteria (Figure 3E), indicating successful FMT engraftment that was stable for 5 weeks.

The microbial community of the Sham group also differed from the initial community structure that was present prior to oral gavage (*q* < 0.05) (Figure 3A), indicating recolonization of the gut irrespective of FMT treatment. Since all animals received antibiotic gut decontamination, this is likely due recovery of endogenous gut microbiota following antibiotic decontamination. The microbial community composition of the Sham group differed from that of the FMT group at all comparative timepoints (*q* < 0.05), indicating no cross-contamination of the control mice from environmental input (Figure 3B). Taxonomic composition of the Sham group showed near total domination by Firmicutes in the following five weeks post antibiotics treatment (Figure 3E), again suggesting recovery of the gut microbiota following antibiotics.

## Discussion

GF mice are ideal models for gut microbiota studies. However, GF models harbour immunological defects, and the feasibility of additional housing requirements render it challenging for many studies. Thus, broad-spectrum antibiotic depletion of the gut microbiota in animals is used as an alternative. However, there are inconsistencies in the method, dose, and duration of treatment regimes. In this study, we determined the optimal duration required for effective gut decontamination as well as frequency of FMT required to achieve effective gut engraftment by donor FMT. We tested for the presence of faecal bacteria at multiple timepoints by multiple modalities using bacterial culture, qPCR, and FISH analysis. Our results showed that remnants of bacteria were present after three days, and that the optimal duration of antibiotic treatment for gut decontamination was seven days. We also showed that three weekly doses of FMT were sufficient for successful engraftment following antibiotic gut decontamination, and that following FMT, bacteria from the donor persisted in animals for the entire five weeks of follow up.

Indeed, antibiotic depletion of microbiota is necessary for the successful engraftment of FMT (37) as microbiota depleted mice have been shown to exhibit equivalent or better engraftment than GF mice (16, 37). However, long periods of antibiotics may lead to unwanted side effects. For instance, 12 days of antibiotic treatment can lead to a significant overgrowth of fungi in mouse faeces (8). Hence, a shorter course of antibiotics is potentially a better approach. Our results supported the previous report indicating that a 7-day course of ampicillin, vancomycin, neomycin, and metronidazole was a viable method to deplete the gut of its native microbiota prior to FMT (8). This was achieved without any observed side-effects, such as dehydration, diarrhoea, or loss of body weight, that may be associated with extended antibiotic treatment.

We observed a reduction in bacteria (both bacterial growth and reduction in bacterial DNA load) within three days with qPCR assessment confirming optimum bacterial DNA depletion was achieved following seven days of antibiotic therapy. This was in concordance with other studies that administered antibiotics via oral gavage or drinking water and observed depletion of the gut microbiota within four days. In this setting, extending the antibiotic regimen beyond seven days did not improve the efficiency of bacteria depletion (8). Notably, the mode of delivery was also important, with similar efficacy when antibiotics were administered via drinking water or twice daily doses via oral gavage. However, a single daily dose via oral gavage did not sufficiently deplete the gut microbiota (8). We opted to deliver the antibiotic cocktail via drinking water, which minimised discomfort and stress to the animals while also being simple and consistent to perform. Taken together, our antibiotic treatment regime combined frequently used antibiotics with a simple delivery method that, when administered for seven days, was highly effective at depleting the gut of bacteria.

We noted that bacteria that remained following antibiotic treatment were not those that were initially abundant in untreated mice, but rather represented residual taxa that were resistant to antibiotic treatment. This supported previous observations that antibiotic treatment exerted a selective pressure on bacterial communities (38), leading to enrichment of communities that were initially scarce. These residual indigenous species had been shown to be able to outcompete a considerable proportion of the human donor microbiota within the first week following FMT (37).

Addressing the question of the frequency and intervals of FMT to achieve successful engraftment is also important. A singular FMT inoculation was shown to elicit no clear improvements in microbial diversity when compared to saline alone (20, 39). Therefore, multiple FMT doses were necessary to maintain a stable engrafted microbiome. Previous concerns were raised that FMT dosing at short intervals, such as twice a week for several weeks, might disturb the newly established ecosystem and impair its equilibrium (22). Our results indicated that three weekly doses of FMT was able to effectively engraft the decontaminated gut with abundances of Firmicutes, Bacteroides, Verrucomicrobia and Proteobacteria similar to that of the donor. Importantly, this similarity in microbiome composition to the human donor sample remained for the entire five weeks of follow up, suggesting that the frequency and interval tested, were successful in achieving stable engraftment.

Despite our results showing good engraftment of donor microbiota, the study does have limitations. A longer follow up period could have determined the length of persistence of successful engraftment in mice. Although there is compelling evidence for the use of antibiotics in successful FMT engraftment, we did not compare our results to mice without antibiotic decontamination, nor to germ free mice due to feasibility.

Although our gut depletion protocol is likely applicable across different groups of mice, other factors need to be taken into consideration when interpreting results utilising gut depletion and subsequent FMT. We have only used young, male C57Bl/6J mice, which are the most widely used inbred, general-purpose strain of mouse as they are a stable strain and easy to breed (40, 41). Nevertheless, the gut microbiota is likely to be different in different strains and in female mice, which may affect the development of disease (42). A number of studies have also demonstrated that the gut microbial structure and diversity changes with age (43–45). However, few studies utilise other mouse strains in gut depletion studies and even fewer have validated the efficacy of gut depletion following antibiotic treatment. Effectiveness of the protocol can easily be confirmed in different strains, ages, and sexes using the same methods in this study. Assessment of the colony-forming units (CFUs) from faecal samples plated in aerobic and/or anaerobic conditions on non-selective media, and qPCR of the 16S rRNA gene allows for culture-independent assessment of gastrointestinal bacterial load. These techniques are easily accessible and reproducible to quickly assess the efficacy of gut microbiota depletion.

The aim of this protocol was to deplete gut bacteria, which is arguably independent of the mouse strain, age and sex. One caveat would be the residual gut microbiota post-antibiotics may be different between mice of different strains, ages and sexes which is an inherent limitation of antibiotic-mediated gut depletion protocols. This would arise from differences in the initial colonisation of the gut microbiota prior to antibiotics treatment. These differences may result in different immune and inflammatory responses (46) and lingering effects of initial host-microbiota interactions cannot be discounted. Depletion of the gut microbiota with broad spectrum antibiotics also changes host immunology and has been associated with altered immune cell populations. For example, secretory IgA and various immune cell types were depleted in the intestine, while in the spleen, dendritic cells and neutrophils were depleted, but basophils were enriched (17) (reviewed by). Thus, care should be taken when planning studies to ensure the gut microbiota is profiled at different timepoints to ensure these factors are taken into consideration. Nevertheless, this protocol is likely applicable to multiple strains of mice of differing ages and sexes, but factors inherent to antibiotic-mediated gut depletion need to be considered.

## Conclusion

We describe a viable and efficient protocol for gut decontamination and microbiota manipulation studies, consisting of seven days of antibiotic administration in drinking water, followed by three weekly doses of faecal transplant into recipient mice. This approach to decontaminate the gut prior to FMT was cost-effective and less invasive, providing an alternative to using GF mice. Standardising methodologies for FMT in animal research are required for the effective translation of pre-clinical findings.

## Data Availability Statement

The original contributions presented in the study are publicly available. This data can be found here: https://www.ncbi.nlm.nih.gov/bioproject/PRJNA765053.

## Ethics Statement

The animal study was reviewed and approved by Animal Care and Ethics Committee, UNSW Sydney.

## Author Contributions

This study was performed in collaboration among all authors. NA designed the study, drafted the manuscript, and performed the statistical analyses. NA and AR collected the animal data. NA, AR, and SKh performed the experimental work. NA and EM performed the microbiome analyses. NA, GH, and AZ analysed the results and supervised the study. SS and SKo revised the manuscript and made significant changes during the review process. All authors contributed to revision of the final manuscript.

## Funding

This study was supported by Sir Owen Glenn grant, RG182137, UNSW. The grant body had no role in the design of the study; in the collection, analyses, or interpretation of data; in the writing of the manuscript, or in the decision to publish the results.

## Conflict of Interest

The authors declare that the research was conducted in the absence of any commercial or financial relationships that could be construed as a potential conflict of interest.

## Publisher's Note

All claims expressed in this article are solely those of the authors and do not necessarily represent those of their affiliated organizations, or those of the publisher, the editors and the reviewers. Any product that may be evaluated in this article, or claim that may be made by its manufacturer, is not guaranteed or endorsed by the publisher.
