# Supplementary material for: Refining a Protocol for Faecal Microbiota Engraftment in Animal Models After Successful Antibiotic-Induced Gut Decontamination
Source: Front Med (Lausanne). 2022 Feb 9;9:770017. doi: 10.3389/fmed.2022.770017 (PMC8864074; doi:10.3389/fmed.2022.770017)
Supplement: Supplementary file 1 [file Data_Sheet_1.pdf]

Refining a protocol for faecal microbiota engraftment in animal models after successful antibiotic-induced gut decontamination

Supplementary Material

Nadia M. Amorim[1], Emily McGovern[1], Anita E. Raposo[1], Saroj Khatiwada[1], SJ Shen[1], Georgina L. Hold[1], Jason M. Behary[1, 2], Emad El-Omar[1, 2], Amany Zekry[1, 2*]

[1]Microbiome Research Centre, St George and Sutherland Clinical School, Australia,
[2]Department of gastroenterology and Hepatology, St George Hospital, Australia

*Correspondence:

Amany Zekry

a.zekry@unsw.edu.au

Keywords: antibiotics, faecal microbiota transplant, microbiome

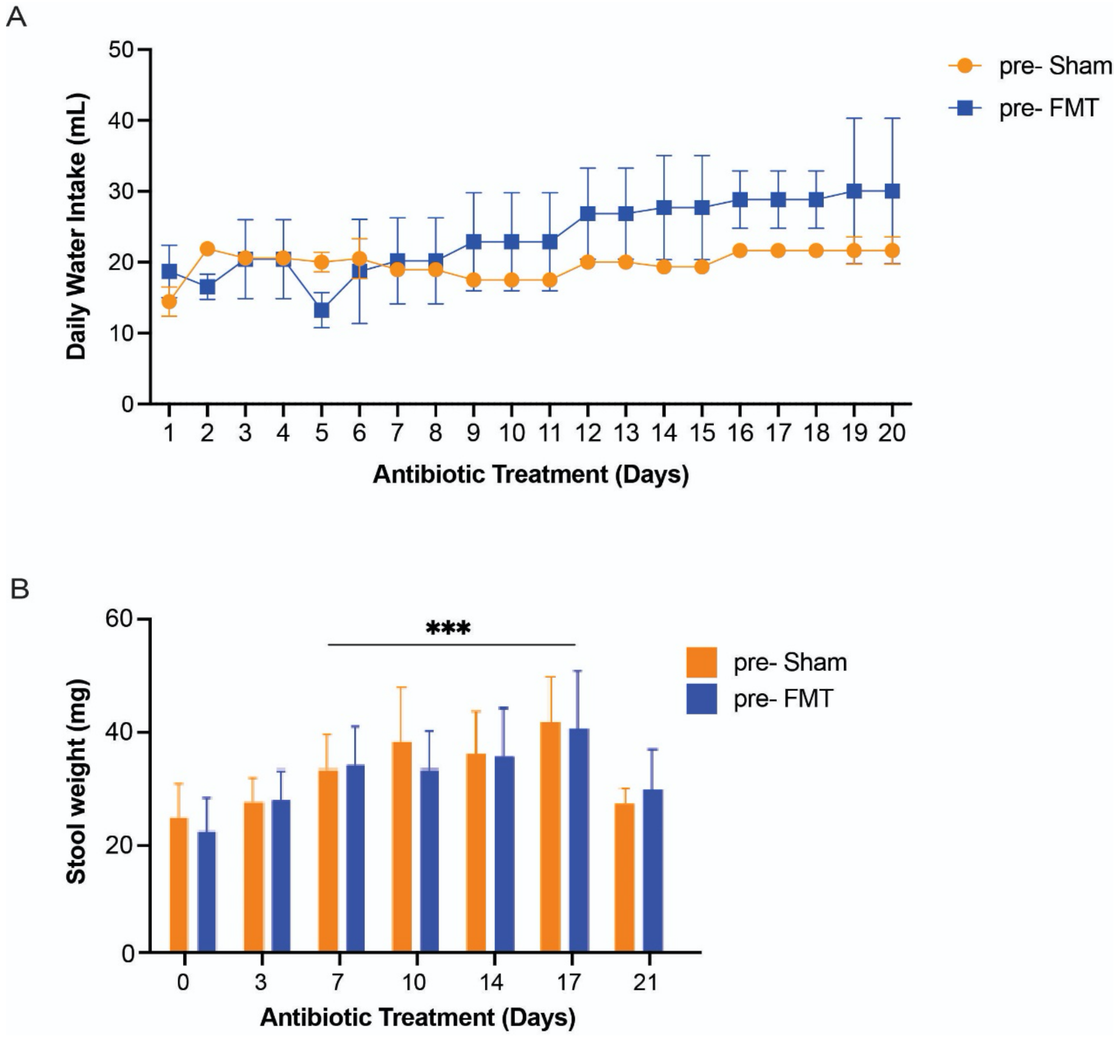

Figure S1. Mouse daily water intake and stool weight during antibiotics treatment period. **(A)** From five weeks of age, mice were given antibiotics in drinking water for 21 days. The amount of water intake was measured daily for animals that would go on to receive saline oral gavage (pre- Sham group) or FMT from human donor stool sample (pre- FMT group). No statistically significant difference in the water intake was observed between the two cohorts of mice. **(B)** At days 0, 3, 7, 10, 14, 17, and 21 of antibiotic treatment, stool samples were collected and weighed (****P* < 0.001 when compared to respective baseline groups, one way ANOVA). Values shown are average ± SD (n =10 mice/group).
